# Tracking development assistance for health from India to low- and middle-income countries, 2009–2020

**DOI:** 10.1371/journal.pone.0277799

**Published:** 2022-12-12

**Authors:** Modhurima Moitra, Nishali K. Patel, Ian Cogswell, Dweep I. Chanana, Emilie Maddison, Kyle Simpson, Hayley Stutzman, Yingxi Zhao, Golsum Tsakalos, Joseph Dieleman, Angela E. Micah

**Affiliations:** 1 Department of Global Health, University of Washington, Seattle, Washington, United States of America; 2 Institute for Health Metrics and Evaluation, University of Washington, Seattle, Washington, United States of America; 3 Department of Health Metrics Sciences, School of Medicine, University of Washington, Seattle, Washington, United States of America; 4 Anchor Group, Geneva, Switzerland; 5 Nuffield Department of Medicine, University of Oxford, Oxford, United Kingdom; University of Glasgow, UNITED KINGDOM

## Abstract

**Background:**

Development assistance for health (DAH) is an important source of financing for health for many low-income and some middle-income countries. Most DAH has predominantly been contributed by high-income countries. However, in the context of economic progress and changing global priorities, DAH contributions from countries of the Global South such as India have gained importance. In this paper, we estimate DAH contributed by India between 2009 and 2020.

**Methods:**

We leveraged data from budgetary documents, databases, and financial reports of the Ministry of External Affairs and multilateral organizations to estimate DAH contributions. The proportions of development assistance that go towards health in major recipient countries were estimated and reported by recipient country and year.

**Results:**

Between 2009 and 2020, DAH contributed by India to bilateral and multilateral partners totaled $206.0 million. South Asian countries including Bangladesh, Bhutan, Nepal, Sri Lanka, and Myanmar received the most DAH from India. DAH contributed relative to DAH received ranged from 1.42% in 2009 to 5.26% in 2018, the latest year with country-level data. Health focus areas prioritized by India included technical training and innovation, health care infrastructure support, and supply of medications and medical equipment.

**Conclusion:**

India is an important development partner to many countries–particularly to those in the South Asian region. India’s DAH allocation strategy prioritizes contributions toward neighboring countries in the South Asia region in several health focus areas. Detailed project-level data are needed to estimate DAH contributions from India with greater precision and accuracy.

## Introduction

Development assistance for health (DAH), which is the financial and non-financial resources transferred through international development agencies to low-income and middle-income countries for the primary purpose of maintaining and improving health, has been a major source of health funding in low- and middle-income countries (LMIC) for the past few decades [1, 2]. In 2020, it made up 25.1% of total health spending in low-income countries [2]. Contributions toward DAH have also risen over time. In 1990, total DAH contributions amounted to $7.7 billion. It has since increased to $40.6 billion as of 2019 and to $52.1 billion in 2020 given the renewed focus on how health systems are financed during COVID-19, growing at a cumulative average growth rate (CAGR) of 6.6% per year [1, 2]. In addition to providing financing for key global health programs related to maternal and child health and communicable diseases, DAH also plays a strategic role in international diplomacy, foreign relations, and economic cooperation [3–5]. Traditionally, most DAH has originated from high-income countries. The Development Assistance Committee (DAC) of the Organisation for Economic Co-operation and Development tracks the majority of official development assistance (ODA). The DAC currently comprises 30 high-income member countries. Estimates of DAH from the Institute for Health Metrics and Evaluation focus on the same set of countries, including assessed contributions to UN agencies and private philanthropy and debt repayments [1, 2, 5]. Since its initiation in 1960, DAC member countries have been some of the largest providers of international aid to LMICs [1, 2, 5].

Historically, some LMICs have also provided development assistance support to other LMICs. More recently, additional countries in the Global South have emerged as major donors of foreign aid given gradual shifts in economic progress and changes in global priorities [6]. Most notably, China’s rapid economic growth in recent years has allowed it to be an influential donor of DAH, with a contribution of $652.3 million in 2017 [7]. An analysis of China’s financial commitments in Africa shows that foreign policy goals (such as United Nations Security Council membership, diplomatic recognition of Taiwan (province of China)) drive Chinese ODA allocations [8].

The role of rising donors and their contributions have important implications for the global DAH landscape. In particular, India has also witnessed considerable economic growth which subsequently increased its geopolitical importance. India’s role as a leader in the Global South-South development cooperation is evident in its founding and participation in several regional collectives, including the Global Network of Export-Import Banks and Development Finance Institutions, the Development Cooperation Forum, and the South Asian Association for Regional Cooperation (SAARC) Development Fund [6–8]. India’s role in global alliances such as BRICS (Brazil, Russia, India, China, and South Africa) is important from a strategic and economic perspective. India, along with the other BRICS member countries, accounts for over 31% of the global gross domestic product (GDP) [9]. The BRICS member countries have also made important aid contributions for diseases such as tuberculosis. Between 2006 and 2013, they provided over 60% of funding for tuberculosis control in 104 countries that account for 94% of total global cases [10].

Given the rising importance and aspirations of India’s influence, particularly in the Global South, it is useful to examine DAH contributed by India to other countries. Several studies have emphasized the impact and significance of overall development assistance contributed by India [7, 8, 11–13]. At present, India does not report development assistance for health provided through standardized mechanisms. Most of India’s development assistance is categorized as “foreign aid” or “overseas development assistance” that includes bilateral grants and loans, lines of credit, and scholarships and technical training via the Indian Technical and Economic Cooperation (I-TECH) program and its corollary Special Commonwealth Assistance for Africa Programme [7, 14].

Estimates of DAH from India are limited in availability given the lack of a formal definition of DAH and sufficient project-level data. Although earlier studies have attempted to provide estimates of overall DAH contributed by India [15–17], there exists a gap in knowledge of DAH contributed by India. In this paper, we generate estimates of DAH provided by India based on available data sources, as well as compare its magnitude against DAH received by India.

## Materials and methods

### Overview

We define DAH from India as bilateral and multilateral aid given in the form of grants as well as educational scholarships. Table 1 provides a list of data sources included in our estimation of DAH. We did not include resources given as lines of credit (concessional loans with subsidized interest rates) contributed by India to LMICs. Although lines of credit are an important form of development assistance, proportions contributed for health purposes were unavailable and therefore were excluded from our estimates. Available data on lines of credit and educational scholarships are reported in, Tables 5 and 6 in S1 File.

**Table 1:** Data sources used for retrospective estimation of DAH from India.

Channel	Data source	Years
*Bilateral*		
Ministry of External Affairs	Annual Outcome Budget [18–23] Grants & Loans, Performance Smart Dashboard [24]	2009–2015
Ministry of Finance	*Excluded from estimation*	
*Multilateral*		
World Health Organization	Annual Contributors, Programme Budget Web Portal, World Health Organization [25]	2014–2020
World Bank IDA	Report from the Executive Directors of the International Development Association to the Board of Directors, World Bank [26]	2018–2019
Gavi, the Vaccine Alliance	Annual Contributions and Proceeds, Gavi [27]	2013–2019
Global Fund to Fight Aids, Tuberculosis and Malaria	Government and Public Donors, The Global Fund, 2009–2020 [28]	2009–2020
United Nations Population Fund	Donor Contributions, United Nations Population Fund [29]	2014–2019

We limited our timeline to the period of 2009 to 2020 to utilize a timeframe that best reflects India’s recent DAH trends and to leverage the most recent year of available data, which was only from 2009 to 2015 for bilateral DAH and 2009 to 2020 for multilateral DAH.

### Estimating DAH from India through bilateral agencies

**Fig 1:**
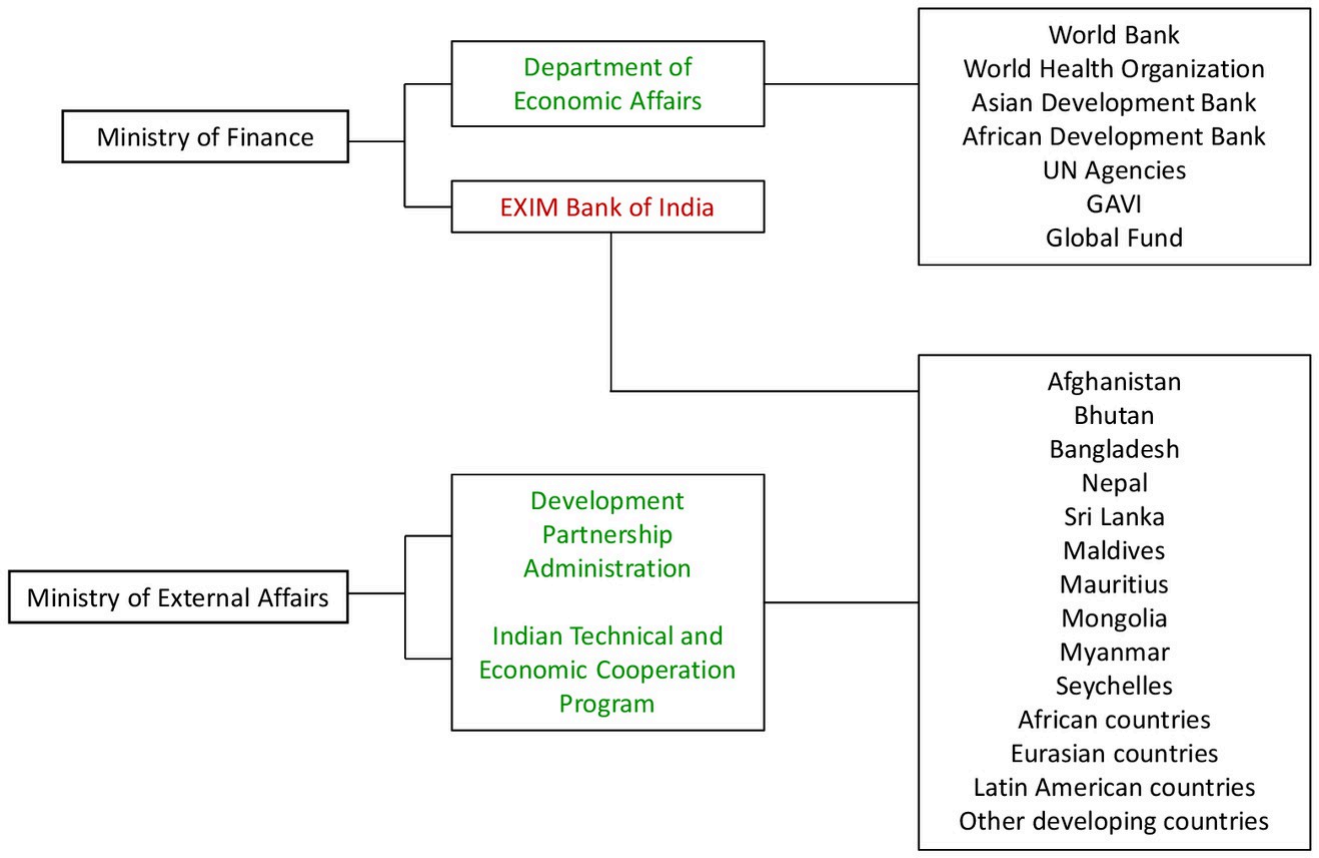
Framework of India DAH dissemination. Note: Green indicates data that have been included in our estimation of DAH, whereas red indicates data that have not been included due to limited availability.

We identified two government agencies–the Ministry of External Affairs and the Ministry of Finance–that are primarily responsible for the disbursement of DAH from India [30, 31] (Fig 1).

Most DAH provided by India through the Ministry of External Affairs is primarily channeled through the Development Partnership Administration (DPA) [32]. Based on our definition of DAH, data on bilateral aid from the Ministry of Finance were excluded as DAH. This is because aid from this government agency is disbursed as concessional loans in the forms of lines of credit through the Export-Import (EXIM) Bank of India [32]. We used annual aggregates of development assistance provided to recipient countries that are reported in the budgetary and financial databases of the Ministry of External Affairs. We utilized these two databases because they reported the most directly comparable annual financial data in terms of scope and duration. From these databases, we compiled disbursements data on bilateral grants for development assistance, overall and specifically for health, for seven South Asian countries (Afghanistan, Bangladesh, Bhutan, Myanmar, Nepal, Seychelles, and Sri Lanka) and four regions (Africa, Latin America, Eurasia, and other developing countries) that reported data between 2009 and 2020.

#### Total development assistance

We extracted the amount of overall development assistance disbursed from the Ministry of External Affairs, available for 2009–2020. Total bilateral development assistance was reported for seven South Asian countries (Afghanistan, Bangladesh, Bhutan, Myanmar, Nepal, Seychelles, and Sri Lanka) and four regions (Africa, Latin America, Eurasia, and other). These values were reported for India’s financial year, which runs from 1 April to 31 March of the following year. To convert values to calendar years, we assigned 75% of the total amount to the first year and 25% for the second year. For instance, if the financial year were 2008/09, 75% of the total DAH for this financial year would be assigned to 2008 and 25% to 2009. Due to a lack of recipient-level information for the four regions, we leveraged country-specific project disbursements data reported in the Ministry of External Affairs’ annual budget reports, available for the years 2009–2015, to disaggregate spending to the country level [18–23]. For each recipient country and year, we calculated the proportion of total development assistance to the region disbursed to the individual recipient country. For instance, the proportion of total development assistance for Africa allocated to Ghana was 26.3% in fiscal year 2012–13 based on the annual financial report. We then assumed that 26.3% of the total development assistance for African countries reported in the Ministry of External Affairs’ database to be the proportion given to Ghana for the fiscal year 2012–13. There were no project-level data available after 2015 to disaggregate regional development assistance by the individual recipient country. To address this, we assumed that the average proportion of total development assistance to each recipient for the period of 2009 to 2015 remained constant for the years with missing data, and we used these proportions to disaggregate total development assistance by the recipient country. For example, the average proportion of total development assistance allocated to Mozambique was 3.7% between 2009 and 2015. We then assumed that 3.7% of the total development assistance for African countries reported in the Ministry of External Affairs’ database to be the proportion given to Mozambique for the years with missing proportions.

#### Total development assistance for health

The proportion of total development assistance allocated to health was estimated using project-level disbursements data from the Ministry of External Affairs’ annual budget reports, available for 2009–2015 [18–23]. From each annual budget report, we extracted project-specific information to identify whether a project disbursement qualified as DAH. Each project disbursement was manually tagged as health-related aid if the description included keywords related to our definition of DAH by two reviewers (NKP and YZ). Discrepancies in health tags between the first and second reviewer were found to be 1.5%. These disagreements were resolved by a third reviewer (AEM) to prevent coding bias. For projects that identified multiple sectors, we divided the total disbursement for that project between the number of identified sectors. For instance, 3.5 crores INR was given “To assist Benin in the Health and Education Sector” in fiscal year 2008–09. In this case, we assigned 50% of this amount as DAH for Benin and 50% for education (excluded from our analyses). Total DAH was then aggregated for each recipient and year. We then calculated a health fraction for each recipient, expressed as a percentage of identified health-related spending to all aid-related spending (Table 1 in S1 File) for the years 2009–2015. This fraction was then multiplied by the total development assistance allocated to the respective recipient country (see “Total development assistance”). For recipients with missing disbursements data within this time series, we imputed health fractions for approximately 9.8% of observations based on a linear rate of change. We assessed the sensitivity of this assumption by imputing both the minimum and maximum fraction observed between 2009 and 2015.

Since there were no project-level data available after 2015, we estimated health fractions using imputation by chained equations with predictive mean matching [33, 34] to impute health fractions for select recipient countries. Between 2016 and 2020, we imputed health fractions for 22 of India’s 67 recipient countries that received DAH for at least five of seven years between 2009 and 2015. We based imputations on covariates relevant to the allocation of DAH, including the year of disbursement, Global Burden of Disease region of the recipient country, total development assistance for the recipient country, GDP per capita, and fragile state index. GDP per capita was normalized prior to imputation modeling, using natural log transformation. Data sources used for covariates are listed in Table 2. For each year included in our analysis, imputation by chained equations was carried out with 20 imputations and 100 iterations [35–37], which resulted in 20 predicted values for each recipient and year. We used the median value of these 20 health fractions as the estimated health fraction for each recipient and year and assessed the variation of the health fraction using the interquartile range (IQR).

**Table 2:** Variables and data sources used for imputing DAH for select recipient countries.

Variable	Data source
Total development assistance	Annual Outcome Budget, 2008/09–2015/16 [18–23] Grants & Loans, Performance Smart Dashboard, 2008/09–2015/16 [24]
Gross domestic product per capita	World Bank, 2009–2020 [38]
Fragile state index	The Fund for Peace, 2009–2020 [39]
Global Burden of Disease region	Institute for Health Metrics and Evaluation, 2019 [40]

### Estimating multilateral DAH from India

In addition to lines of credit provided through the EXIM Bank, the Ministry of Finance funds multilateral contributions to the World Bank, World Health Organization, Asian Development Bank, African Development Bank, Gavi, the Global Fund, and United Nations agencies. There were limited data available on contributions to these multilateral agencies. Available data on these multilateral contributions from India were extracted from annual financial and replenishment reports of multilateral agencies [25–29]. Disbursements data on multilateral grants, including assessed and voluntary funding, to the health sector were included in the overall estimate of DAH contributions made by India from 2009 to 2020.

### Aggregating India’s total DAH contribution

For bilateral and multilateral aid, we converted values in Indian rupees (INR) into US dollars (USD) based on year-specific exchange rates extracted from the OECD exchange rate database [41]. We deflated disbursements to constant 2020 USD using the International Monetary Fund deflator series [42]. India’s total DAH contribution was aggregated by adding up bilateral and multilateral contributions. All analyses were conducted using R version 4.0.5 (2021-04-30).

### Comparing DAH from India

In addition to the data sources above, we extracted DAH given to India for 2009–2018 from the DAH database of the Institute for Health Metrics and Evaluation (IHME) [43]. These data were used to compare the DAH contributed by India and the DAH received by India.

## Results

**Fig 2:**
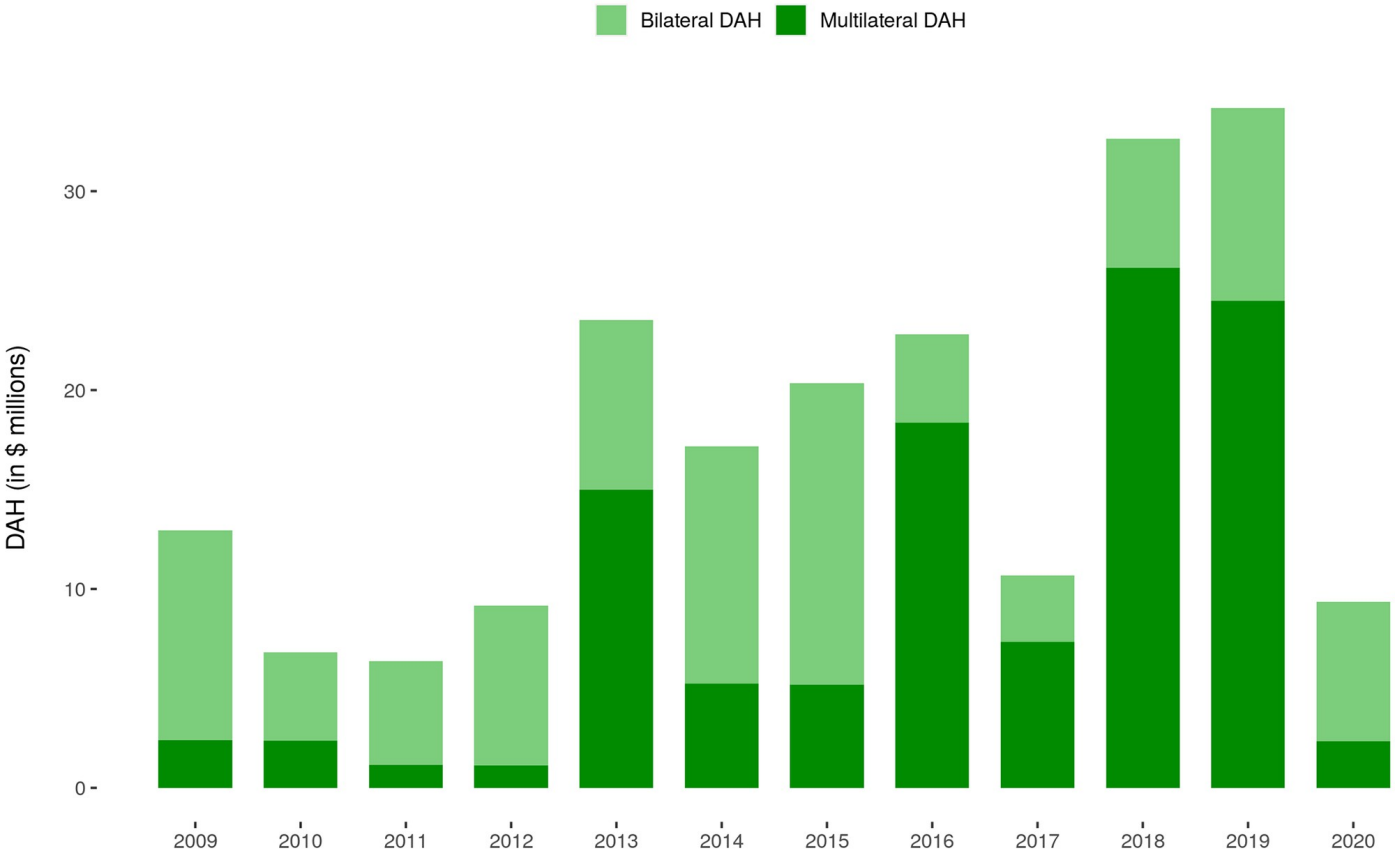
Estimated annual DAH contributed by India, 2009–2020. Note: Annual DAH reported here includes bilateral grants and multilateral contributions. Multilateral contributions included both annual contributions and replenishments.

Fig 2 represents the total estimated DAH contributions made by India, disaggregated by bilateral and multilateral contributions. Between 2009 and 2020, total DAH contributed by India to bilateral and multilateral partners was US$206.0 million. Multilateral contributions made up the majority of DAH in 2013 and 2016 to 2019.

Table 3 reports bilateral contributions disaggregated by recipient country for 2009 to 2015. DAH contributions are disaggregated (or shown separately) as these varied considerably across recipients. Collectively, DAH contributions from India to bilateral recipients were $65.2 million between 2009 and 2015. Contributions to Myanmar ranged from $0.94 million in 2012 to $3.7 million in 2015, whereas contributions to Sri Lanka ranged from $0.07 million in 2009 to $3.0 million in 2014 and $1.9 million in 2015. Recipient countries with the highest proportion of DAH contributions from India included Afghanistan, Nepal, Myanmar, Sri Lanka, the Maldives, and The Gambia. Based on project-level data available for 2009–2015, DAH contributions by India have been primarily allocated to projects that focus on health care infrastructure support, supply of medications and medical equipment, and technical training and innovation.

**Table 3:** Estimated annual DAH (USD) contributed from by India disaggregated by year and select recipient country (2009–2015). Red and green represent high and low contributions to the recipient country, respectively.

Recipient country	2009	2010	2011	2012	2013	2014	2015
Afghanistan	2,390,511	575,906	3,511,322	1,176,159	370,429	3,792,280	5,622,645
Angola						4,035	2,196
Armenia						55,025	20,440
Bangladesh					181,090	789,046	54,051
Benin	522,555	151,916	31,663	7,540	3,052	5,347	11,388
Bhutan	223,171	1,046,581	298,489				
Botswana				37,695	10,499	3,975	2,049
Burkina Faso	254,275	65,334	36,951	7,523	3,049	5,281	10,749
Burundi						3,931	1,844
Cambodia	405,533	119,377					
Cameroon						4,035	2,196
Cape Verde	235,906	61,314	22,171	7,540	3,039	5,379	11,388
Central African Republic						3,989	2,089
Chad						4,043	2,210
Comoros						4,050	2,210
Côte d’Ivoire	232,870	65,334	30,394	7,540	3,052	5,347	11,416
Democratic Republic of Congo	169,064					3,991	2,093
Equatorial Guinea						4,043	2,210
Eritrea						4,050	2,210
Eswatini						4,043	2,210
Ethiopia	157,461	40,696	3,714	3,166	5,798	4,001	6,127
Fiji						91,732	48,771
Gabon						4,030	2,175
Gambia	235,906	65,334	87,227	2,035,953	618,728	5,295	11,667
Ghana	235,906	65,153	26,809	7,617	3,364	5,182	14,240
Guinea	235,906	65,334	30,394	7,540	3,052	5,347	11,388
Guinea-Bissau	235,906	65,334	30,394	7,540	3,052	5,347	11,388
Kazakhstan						37,099	14,030
Kenya					12,422	7,745	3,539
Kyrgyzstan						37,169	14,259
Lesotho						4,043	2,210
Liberia	246,984	69,880	20,655	7,556	3,063	132,460	123,858
Madagascar						3,947	1,949
Malawi			73,771	183,004	44,761	205,309	671,904
Maldives				829,929	465,459	956,563	1,349,594
Mali	235,906	65,334	30,394	7,540	3,052	5,337	11,314
Mauritania	235,906	65,334	30,394	7,540	3,052	5,242	10,339
Mozambique						4,048	2,213
Myanmar				943,550	3,071,408	1,377,835	3,738,078
Namibia					12,436	7,742	2,301
Nepal	2,614,601	1,396,521	1,123,108	1,724,639	1,652,323	1,016,241	1,338,933
Nicaragua					23,478	7,352	
Niger	235,906	65,334	30,394	7,540	3,052	5,347	11,388
Nigeria	235,906	65,334	38,752	7,519	3,052	5,152	9,874
Philippines	11,635						
Republic Of Congo	151,027	39,371	35,941	30,395	41,991	3,991	2,261
Rwanda						3,990	2,241
Samoa	123,594	12,318					
São Tomé and Príncipe						4,043	2,210
Senegal	230,202	63,339	37,044	7,536	3,046	9,394	17,428
Sierra Leone	235,906	65,334	30,394	7,540	3,052	5,347	11,388
Somalia						4,050	2,247
South Africa					12,422	7,849	6,092
South Sudan						4,050	2,210
Sri Lanka	71,849	251,940	715,312	975,353	2,017,447	2,969,429	1,809,885
Sudan						4,041	2,192
Tajikistan						35,613	14,286
Tanzania						3,942	5,293
Togo	276,564	65,334	30,394	7,540	3,052	5,335	11,297
Turkmenistan						37,460	14,229
Tuvalu	124,462					139,347	69,884
Uganda						3,981	2,047
Uzbekistan						36,238	13,810
Zambia						4,025	2,175
Zimbabwe						3,907	1,992

Table 4 reports estimated bilateral contributions for 22 recipient countries for 2016 to 2020. Collectively, DAH contributions from India to select bilateral recipients was $31.5 million. Contributions to Nepal ranged from $0.89 million in 2016 to $4.3 million in 2020, whereas contributions to Afghanistan ranged from $1.6 million in 2016 to $1.2 million in 2020. Recipient countries with the highest proportion of DAH contributions from India between 2016 and 2020 included Nepal, Afghanistan, Sri Lanka, and Seychelles.

**Table 4:** Estimated annual DAH (USD) contributed by India disaggregated by year and select recipient countries (2016–2020). Red and green represent high and low contributions to the recipient country, respectively.

Recipient Country	2016	2017	2018	2019	2020
Afghanistan	1,620,098	1,266,426	1,486,563	1,348,922	1,194,737
Benin	64,501	74,649	149,589	277,785	245,257
Burkina Faso	31,488	11,829	129,879	26,931	66,565
Cape Verde	18,917	10,976	19,943	119,529	41,492
Côte d’Ivoire	17,249	13,168	22,287	27,971	20,324
Ethiopia	18,360	41,049	9,890	19,708	16,286
Gambia	128,498	26,282	85,290	449,499	175,695
Ghana	124,916	177,219	282,725	321,188	305,079
Guinea	19,512	12,407	16,205	27,898	8,895
Guinea-Bissau	23,776	10,568	16,205	20,429	10,318
Liberia	19,010	16,989	18,335	149,948	22,330
Malawi	91,119	102,672	63,644	246,462	58,038
Mali	13,979	31,151	11,379	22,563	27,030
Mauritania	21,400	10,907	23,959	30,758	18,892
Nepal	894,429	1,083,549	3,123,156	5,085,065	4,268,907
Niger	15,663	9,857	65,021	23,880	14,778
Nigeria	39,248	21,597	27,708	35,616	39,164
Republic of Congo	26,546	11,739	13,352	34,568	16,045
Senegal	9,905	13,452	68,865	36,237	162,516
Seychelles	244,132	790,134	685,621	108,062	313,874
Sierra Leone	65,207	37,706	50,265	113,926	39,649
Sri Lanka	511,089	284,320	489,216	628,041	253,857
Togo	15,341	10,349	14,863	22,146	13,011

**Fig 3:**
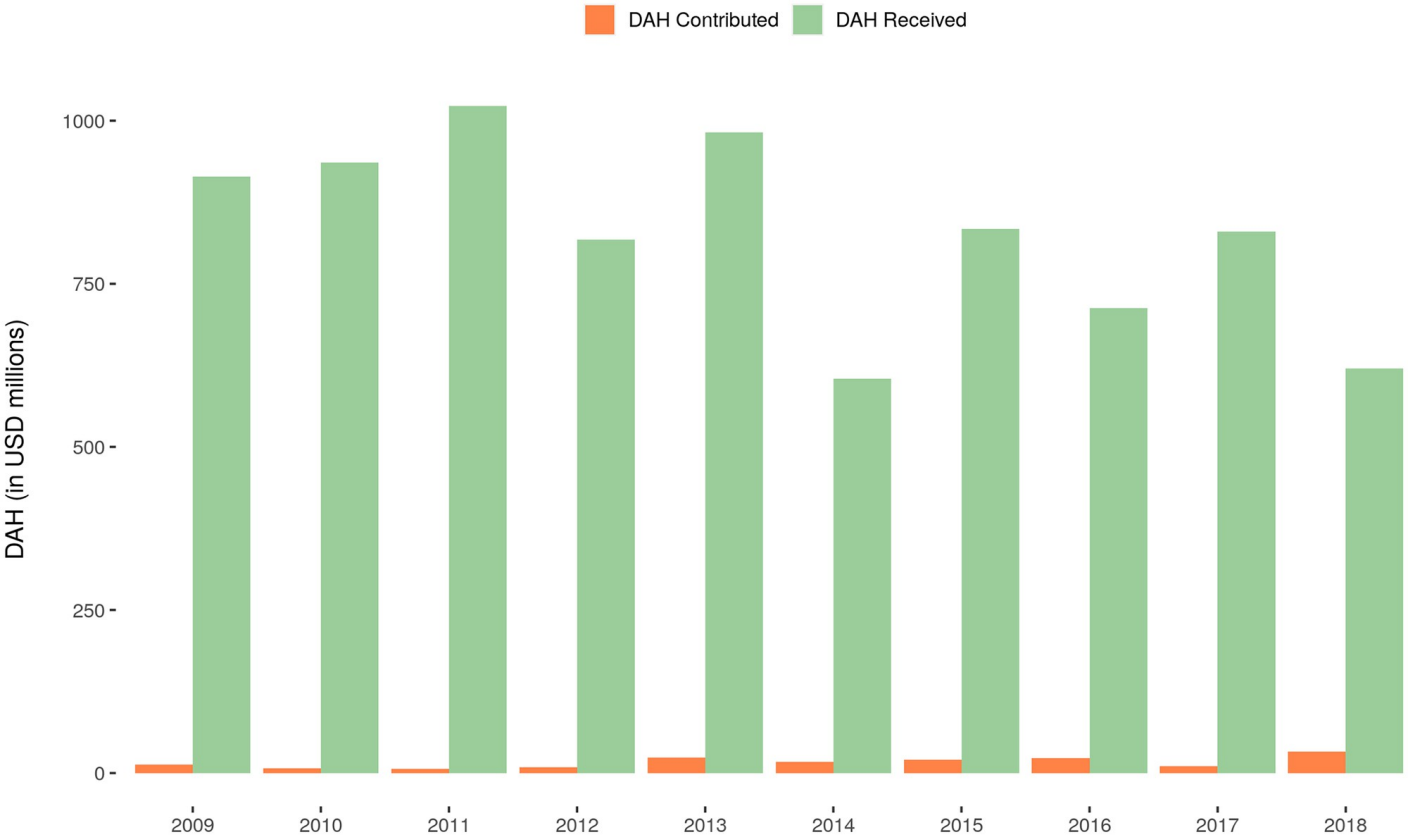
Bilateral DAH received and contributed by India, 2009–2018. Note: This figure uses data on DAH received by India from Micah et al. (2021) which reports DAH received by India up to 2018.

Fig 3 shows the total amount of bilateral DAH received and contributed by India. Bilateral DAH contributed relative to bilateral DAH received was 1.42% in 2009 and increased to 5.26% in 2018.

There were limited data on DAH channeled via multilateral agencies. Most contributions to multilateral agencies were made intermittently between 2016 and 2019, either in the form of annual contributions or replenishments. The institutions and organizations were identified as having available estimates on DAH contributions channeled through the Ministry of Finance included World Bank IDA, Gavi, the Global Fund, UNFPA, and the World Health Organization. Contributions to Gavi primarily included vaccine supplies and delivery. Contributions to the Global Fund were toward malaria, HIV/AIDS, and tuberculosis. UNFPA contributions were made toward sexual and reproductive health. World Bank IDA contributions were made toward health projects; however, there was no information on specific areas of health improvement. Detailed contributions by health priority areas were available for WHO [25]. This included tuberculosis, polio, health systems strengthening, epidemic and pandemic prevention, health technologies, and reproductive, maternal, and child health. Multilateral contributions by agency are reported separately in S1 Fig.

## Discussion

This paper highlights DAH contributions made by India between 2009 and 2020, leveraging development assistance data from official government sources and financial statements of key multilateral partners. To our knowledge, this study is the first effort to comprehensively estimate DAH contributions made by India beyond 2010.

Total DAH contributions from India were $206.0 million inclusive of both bilateral and multilateral contributions. The decrease in DAH between 2016 and 2017 may have been associated with increases in overall lines of credit channeled via the Ministry of Finance, which were not included in this study [15]. This may be reflective of new policy shifts in how development assistance is channeled [15]. There was also a sharp decrease in DAH in 2020, similar to overall development assistance [24], which may indicate shifts in funding priorities in response to COVID-19 within India.

Bilateral contributions predominantly focus on health systems strengthening, health infrastructure, and human resources support, similar to multilateral contributions. DAH contributed relative to DAH received increased from 2009 through 2018. While the overall increase in DAH contributed relative to DAH received may be small, it is suggestive of a gradual decline in reliance on received DAH and a growing commitment to increase DAH contributed to other countries.

Our estimates are consistent with recently reported data [16]. However, several reported estimates of DAH contributed by India vary widely in the existing literature. For instance, total foreign health aid by India in 2007–2008 is approximated to be $226 million in one study [15]. Other estimates on sectoral allocations of total development assistance estimate about 7.5% to health-related activities between 2008 and 2010 [17]. Variation in estimates may be due to differences in how DAH is defined, which may pose challenges to facilitating consistent comparisons between estimates. DAH as defined in this study does not include water and sanitation, food assistance, humanitarian aid, or aid for poverty alleviation. It also does not include loans that are not concessional in nature. Therefore, it is plausible that these estimates may be more conservative than existing ones.

India’s overall development assistance contributions have consistently reflected both a regional focus, with most assistance being allocated to neighboring South Asian countries, and a gradually expanding global focus with allocations to Africa, Central Asia, Latin America, and Southeast Asia (Fig 1, Tables 2 and 3) [8, 16, 44]. India is a key member of the BRICS alliance, which will likely continue to be an important forum to promote international development and strengthen collaborations with its member countries [9]. India recently chaired the 2021 BRICS Summit and used this platform to consolidate regional influence in multiple spheres, including promoting global health, traditional medicine, and digital health [45]. In terms of global health priority areas, India’s DAH is likely to continue its focus on technical training, innovation, education and scholarships, medication supplies, and infrastructure that will contribute to health systems and human resource strengthening at the local level in recipient countries based on historical data. This form of assistance has been received positively as it focuses on capacity building at the local level without undermining local institutions or reducing a recipient country’s competitiveness [7]. Economic and political interests may also drive India’s current and future development assistance strategy. The political and economic motivations of expanded development assistance efforts, including aligned interests in trade, opening up of new markets, strengthening economic relations, and diplomatic influence, have been discussed by several experts [6–8, 46, 47]. Changes in trends of development assistance contributions have also been observed in terms of country allocations as well as budgetary allocations. Recent analyses of the Indian Union budget have observed fluctuations in bilateral allocations to South Asian countries overall, with noted decreases in allocations to Bangladesh, Afghanistan, and Sri Lanka between 2013–2014 and 2016–2017 [15]. Empirical analyses show that factors such as United Nations General Assembly voting alignment, geographic proximity of recipients, and similarity of development profiles are important indicators that may be potential drivers of India’s aid allocation strategy [30].

The data sources used in this analysis provide the most comprehensive assessment of India’s DAH contributions. However, there are clear limitations. To start with, we heavily relied on government annual budget reports to estimate the proportion of development assistance allocated to the health sector. This source, despite being official, reports descriptions of development assistance projects inconsistently across years and frequently lacks granularity at the project level to determine whether aid projects should be assigned to the health sector. For example, some projects did not have a description or were provided generally as multisectoral aid; in such instances, we did not include these projects in our estimates of DAH. Second, we imputed health fractions for approximately 9.8% of observations based on a linear rate of change for recipients with missing disbursements data between 2009 and 2015. Imputations were only applied for countries missing data points between three years. This uncertainty may have inflated our estimates slightly, specifically in 2011 by 9.2%. We included the imputed missing values and results of this sensitivity analysis in Table 3 in S1 File to examine the robustness of our estimates for bilateral DAH.

Third, project-level data were only available for the years 2009–2015, and as such, we used imputation with predictive mean matching to predict the proportion allocated to health for the years 2016–2020 for recipients with a near-complete time series of data. We are aware that the proportion allocated to health is affected by other factors which are difficult to model (e.g., national funding priorities), and using predicted values could easily influence our final estimates for the most recent years. This uncertainty could influence our final estimate: for example, the IQR for overall DAH contributed by India between 2016 and 2020 was $20.5 million–$50.7 million. Further details of this sensitivity analysis are included in Table 4 in S1 File. Additionally, we chose to predict DAH for 22 recipient countries only due to lack of consistent data for all bilateral recipients, which may underestimate our results for bilateral DAH between 2016 and 2020 as other recipients are likely excluded. Nonetheless, we believe that these methods and data are the best available information we could use to help us understand the total DAH contributed by India. Finally, due to the limitations of sparse data on the amount of development assistance from India that goes toward health, we chose to estimate India’s DAH only for select forms of aid (e.g., bilateral and multilateral grants) at the national level. Overall estimates of multilateral DAH contributions were available for only select global health institutes, and therefore no detailed estimates by recipient country could be made. Other forms of assistance such as lines of credit (channeled through Exim Bank) are available in varying forms and make up a significant portion of India’s overall aid budget [48], but we lacked information on the approximate proportion of lines of credit allocated to health. Our estimates also do not include data on DAH from private sources such as philanthropic giving, and corporate social responsibility contributions are predominantly domestic in scope. As a result, estimates of DAH contributed by India reported here are likely underestimates of the actual DAH that India may have contributed during this time period. Future efforts to estimate DAH contributed by India will require more detailed project-level data for additional countries and years in order to obtain more precise estimates.

## Conclusions

This study reported estimated DAH contributions from India to recipient countries and multilateral organizations between 2009 and 2020. India’s DAH contribution reached a total of $206.0 million and heavily focused on providing support to neighboring South Asian countries for health systems strengthening, infrastructure support, infectious diseases, and reproductive health. The data limitations of this analysis highlight the challenges that arise from limited data availability, namely a lack of granular project-level data after 2015 and financial information to estimate the proportion of development assistance given in the form of lines of credit for DAH through Exim Bank. Further, we used imputation by chained equations to generate out-of-sample predictions of DAH for the years 2016–2020 for a subset of recipient countries, which may not reflect shifting policy priorities toward or away from health. It remains important to obtain detailed data from emerging donors such as India in order to track its DAH contributions with greater precision. As such, official centralized databases that publish greater detail on development projects will become increasingly important to improve tracking of global health financing to LMICs as well as cooperation between local and regional donors.

## Supporting information

S1 File Contains all supporting tables. (DOCX)

S1 Fig DAH contributions by India to multilateral agencies, 2016–2019. (Note: Multilateral contributions shown here include both annual and replenishment amounts).
(TIF)
