# Supplementary material for: Tracking development assistance for health from India to low- and middle-income countries, 2009–2020
Source: PLoS One. 2022 Dec 12;17(12):e0277799. doi: 10.1371/journal.pone.0277799 (PMC9744314; doi:10.1371/journal.pone.0277799)
Supplement: S1 File — (DOCX) [file pone.0277799.s001.docx]

Supplementary Appendix 1

Title: Tracking development assistance for health from India to low and middle-income countries, 2009-2020

Authors: Modhurima Moitra, PhD*^1^, Nishali K. Patel, MSc*^2, 3^, Ian Cogswell, BS^2^, Dweep I. Chanana^4^, Emilie Maddison, BS^2^, Kyle Simpson, BS^2^, Hayley Stutzman, BA^2^, Yingxi Zhao, MPH^2,5^, Golsum Tsakalos, MS^2^, Joseph Dieleman, PhD^2,3^, Angela E. Micah, PhD^2,3^

* Joint first authors

Affiliations: 1 Department of Global Health, University of Washington, Seattle, WA, USA

                     2 Institute for Health Metrics and Evaluation, University of Washington, Seattle, WA, USA

         3 Department of Health Metrics Sciences, University of Washington, School of Medicine, Seattle, WA, USA

                     4 Anchor Group

5 Nuffield Department of Medicine, University of Oxford, Oxford, UK

Corresponding: Nishali K. Patel, MSc

patelnk@uw.edu

University of Washington, Seattle, USA

Objective: The purpose of this study was to generate estimates for development assistance for health (DAH) contributed by India and other Southeast Asian countries to low and middle-income countries. We obtain estimates of DAH contributed by India from government data sources and literature review. We report estimates of DAH contributed by Southeast Asian countries using estimates generated by the Institute for Health Metrics and Evaluation.

List of Appendix Contents:

S1 Table 1: DAH proportions for India, 2009 – 2015

S1 Table 2: Estimated DAH proportions for India, 2015/16 – 2020/21

S1 Table 3: Sensitivity analysis for DAH estimated for 2009 and 2015

S1 Table 4: Interquartile range for predicted DAH, 2016 – 2020

S1 Table 5: List of countries receiving Lines of Credit from India

S1 Table 6: Estimation of scholarships provided by India as part of DAH contributions

S1 Table 1: DAH proportions for India, 2009 – 2015

| **Recipient Country** | **Year** | | | | | | |
| --- | --- | --- | --- | --- | --- | --- | --- |
|  | **2009** | **2010** | **2011** | **2012** | **2013** | **2014** | **2015** |
| Afghanistan | 0.042 | 0.009 | 0.054 | 0.014 | 0.004 | 0.033 | 0.041 |
| Angola |  |  |  |  |  | 0.010 | 0.004 |
| Armenia |  |  |  |  |  | 0.264 | 0.089 |
| Bangladesh |  |  |  |  | 0.002 | 0.016 | 0.002 |
| Benin | 0.196 | 0.152 | 0.085 | 0.018 | 0.004 | 0.014 | 0.020 |
| Bhutan | 0.001 | 0.003 | 0.001 |  |  |  |  |
| Botswana |  |  |  | 0.073 | 0.014 | 0.007 | 0.003 |
| Burkina Faso | 0.310 | 0.146 | 0.081 | 0.017 | 0.004 | 0.010 | 0.017 |
| Burundi |  |  |  |  |  | 0.004 | 0.002 |
| Cambodia | 0.344 | 0.288 |  |  |  |  |  |
| Cameroon |  |  |  |  |  | 0.010 | 0.004 |
| Cape Verde | 0.293 | 0.069 | 0.043 | 0.018 | 0.003 | 0.012 | 0.020 |
| Central African Republic |  |  |  |  |  | 0.008 | 0.003 |
| Chad |  |  |  |  |  | 0.011 | 0.004 |
| Comoros |  |  |  |  |  | 0.010 | 0.004 |
| Côte d'Ivoire | 0.278 | 0.146 | 0.082 | 0.018 | 0.004 | 0.014 | 0.020 |
| Democratic Republic of Congo | 0.533 |  |  |  |  | 0.008 | 0.003 |
| Equatorial Guinea |  |  |  |  |  | 0.011 | 0.004 |
| Eritrea |  |  |  |  |  | 0.010 | 0.004 |
| Eswatini |  |  |  |  |  | 0.011 | 0.004 |
| Ethiopia | 0.324 | 0.121 | 0.007 | 0.007 | 0.007 | 0.007 | 0.005 |
| Fiji |  |  |  |  |  | 0.375 | 0.165 |
| Gabon |  |  |  |  |  | 0.010 | 0.004 |
| Gambia | 0.293 | 0.146 | 0.192 | 0.238 | 0.184 | 0.010 | 0.008 |
| Ghana | 0.293 | 0.142 | 0.071 | 0.001 | 0.001 | 0.004 | 0.007 |
| Guinea | 0.293 | 0.146 | 0.082 | 0.018 | 0.004 | 0.014 | 0.020 |
| Guinea-Bissau | 0.293 | 0.146 | 0.082 | 0.018 | 0.004 | 0.014 | 0.020 |
| Kazakhstan |  |  |  |  |  | 0.108 | 0.032 |
| Kenya |  |  |  |  | 0.015 | 0.014 | 0.006 |
| Kyrgyzstan |  |  |  |  |  | 0.245 | 0.036 |
| Lesotho |  |  |  |  |  | 0.011 | 0.004 |
| Liberia | 0.145 | 0.097 | 0.056 | 0.014 | 0.004 | 0.178 | 0.081 |
| Madagascar |  |  |  |  |  | 0.005 | 0.003 |
| Malawi |  |  | 0.111 | 0.225 | 0.054 | 0.176 | 0.294 |
| Maldives |  |  |  | 0.220 | 0.200 | 0.254 | 0.177 |
| Mali | 0.293 | 0.146 | 0.082 | 0.018 | 0.004 | 0.014 | 0.020 |
| Mauritania | 0.293 | 0.146 | 0.082 | 0.018 | 0.004 | 0.008 | 0.016 |
| Mauritius |  |  |  |  |  | 0.002 | 0.001 |
| Mozambique |  |  |  |  |  | 0.011 | 0.004 |
| Myanmar |  |  |  | 0.049 | 0.115 | 0.069 | 0.204 |
| Namibia |  |  |  |  | 0.016 | 0.015 | 0.001 |
| Nepal | 0.074 | 0.041 | 0.031 | 0.036 | 0.027 | 0.019 | 0.027 |
| Nicaragua |  |  |  |  | 0.213 | 0.082 |  |
| Niger | 0.293 | 0.146 | 0.082 | 0.018 | 0.004 | 0.014 | 0.020 |
| Nigeria | 0.293 | 0.146 | 0.081 | 0.017 | 0.004 | 0.003 | 0.006 |
| Philippines | 0.048 |  |  |  |  |  |  |
| Republic of Congo | 0.459 | 0.130 | 0.100 | 0.076 | 0.058 | 0.008 | 0.002 |
| Rwanda |  |  |  |  |  | 0.007 | 0.002 |
| Samoa | 0.328 | 0.085 |  |  |  |  |  |
| São Tomé and Príncipe |  |  |  |  |  | 0.011 | 0.004 |
| Senegal | 0.264 | 0.107 | 0.059 | 0.011 | 0.002 | 0.014 | 0.018 |
| Seychelles |  | 0.076 | 0.365 | 0.008 | 0.009 | 0.010 | 0.007 |
| Sierra Leone | 0.293 | 0.146 | 0.082 | 0.018 | 0.004 | 0.014 | 0.020 |
| Somalia |  |  |  |  |  | 0.010 | 0.003 |
| South Africa |  |  |  |  | 0.015 | 0.019 | 0.010 |
| South Sudan |  |  |  |  |  | 0.010 | 0.004 |
| Sri Lanka | 0.005 | 0.014 | 0.023 | 0.023 | 0.031 | 0.037 | 0.026 |
| Sudan |  |  |  |  |  | 0.010 | 0.004 |
| Tajikistan |  |  |  |  |  | 0.083 | 0.016 |
| Tanzania |  |  |  |  |  | 0.005 | 0.007 |
| Togo | 0.330 | 0.146 | 0.082 | 0.018 | 0.004 | 0.013 | 0.020 |
| Turkmenistan |  |  |  |  |  | 0.184 | 0.029 |
| Tuvalu | 0.194 |  |  |  |  | 0.693 | 0.256 |
| Uganda |  |  |  |  |  | 0.007 | 0.003 |
| Uzbekistan |  |  |  |  |  | 0.091 | 0.049 |
| Zambia |  |  |  |  |  | 0.010 | 0.004 |
| Zimbabwe |  |  |  |  |  | 0.003 | 0.001 |

Note: values in red are imputed using an average linear rate of change between two or three years for select countries with a near-complete time series.

S1 Table 2: Sensitivity analysis for DAH between 2010 and 2015

| **Year** | **Total DAH (USD $),**  **adjusted for missing health proportions** | **DAH (USD $),**  **no adjustment** | **Difference** | **Percentage Difference (%)** |
| --- | --- | --- | --- | --- |
| 2009 | 10,565,419.85 | 10,565,419.85 | - | 0.00 |
| 2010 | 4,612,987.95 | 4,361,048.41 | 251,940 | 5.46 |
| 2011 | 6,306,078.59 | 5,725,714.66 | 580,364 | 9.20 |
| 2012 | 8,052,997.40 | 8,019,436.38 | 33,561 | 0.42 |
| 2013 | 8,586,775.14 | 8,538,986.07 | 47,789 | 0.56 |
| 2014 | 11,927,482.15 | 11,927,482.15 | - | 0.00 |
| 2015 | 15,170,306.66 | 15,170,306.66 | - | 0.00 |

S1 Table 3: Predicted DAH proportions for India, 2016 – 2020

| **Recipient Country** | **Year** | | | | |
| --- | --- | --- | --- | --- | --- |
|  | **2016** | **2017** | **2018** | **2019** | **2020** |
| Afghanistan | 0.023 | 0.016 | 0.015 | 0.021 | 0.011 |
| Benin | 0.029 | 0.016 | 0.046 | 0.018 | 0.065 |
| Burkina Faso | 0.020 | 0.081 | 0.082 | 0.039 | 0.028 |
| Cape Verde | 0.019 | 0.047 | 0.020 | 0.020 | 0.038 |
| Côte d'Ivoire | 0.023 | 0.018 | 0.017 | 0.021 | 0.016 |
| Ethiopia | 0.020 | 0.019 | 0.037 | 0.020 | 0.020 |
| Gambia | 0.114 | 0.014 | 0.020 | 0.069 | 0.055 |
| Ghana | 0.055 | 0.076 | 0.082 | 0.020 | 0.064 |
| Guinea | 0.020 | 0.018 | 0.018 | 0.019 | 0.018 |
| Guinea-Bissau | 0.016 | 0.062 | 0.019 | 0.019 | 0.020 |
| Liberia | 0.072 | 0.020 | 0.020 | 0.020 | 0.019 |
| Malawi | 0.144 | 0.084 | 0.065 | 0.046 | 0.173 |
| Mali | 0.016 | 0.030 | 0.016 | 0.019 | 0.020 |
| Mauritania | 0.018 | 0.030 | 0.018 | 0.043 | 0.018 |
| Nepal | 0.025 | 0.018 | 0.023 | 0.027 | 0.019 |
| Niger | 0.019 | 0.072 | 0.018 | 0.041 | 0.040 |
| Nigeria | 0.021 | 0.017 | 0.020 | 0.020 | 0.023 |
| Republic of Congo | 0.019 | 0.033 | 0.019 | 0.025 | 0.020 |
| Senegal | 0.044 | 0.062 | 0.019 | 0.018 | 0.019 |
| Seychelles | 0.015 | 0.019 | 0.023 | 0.020 | 0.021 |
| Sierra Leone | 0.109 | 0.077 | 0.107 | 0.068 | 0.164 |
| Sri Lanka | 0.014 | 0.021 | 0.023 | 0.023 | 0.014 |
| Togo | 0.020 | 0.020 | 0.020 | 0.017 | 0.032 |

S1 Table 4: Interquartile range for predicted DAH, 2016 – 2020

| Recipient | 2016 | | 2017 | | 2018 | | 2019 | | 2020 | |
| --- | --- | --- | --- | --- | --- | --- | --- | --- | --- | --- |
|  | 25th | 75th | 25th | 75th | 25th | 75th | 25th | 75th | 25th | 75th |
| Afghanistan | 577,323 | 2,180,629 | 1,119,145 | 1,736,224 | 1,387,633 | 2,023,743 | 747,459 | 1,570,622 | 926,923 | 1,885,390 |
| Benin | 13,739 | 169,259 | 17,066 | 221,081 | 18,667 | 386,003 | 34,927 | 573,104 | 54,424 | 325,494 |
| Burkina Faso | 10,370 | 136,248 | 4,138 | 90,217 | 12,810 | 141,851 | 16,899 | 188,462 | 10,423 | 145,029 |
| Cape Verde | 15,654 | 90,995 | 3,781 | 13,967 | 7,654 | 144,303 | 29,887 | 214,249 | 8,961 | 164,953 |
| Cote d'Ivoire | 6,097 | 43,580 | 4,585 | 21,233 | 11,784 | 67,579 | 15,460 | 50,913 | 3,169 | 56,512 |
| Ethiopia | 5,157 | 63,791 | 3,883 | 94,486 | 3,701 | 19,465 | 5,495 | 107,034 | 6,419 | 65,741 |
| Gambia | 27,964 | 343,490 | 8,210 | 238,125 | 28,876 | 207,512 | 160,809 | 718,473 | 43,659 | 307,211 |
| Ghana | 32,347 | 449,885 | 31,124 | 323,154 | 52,904 | 640,688 | 214,143 | 762,228 | 131,527 | 566,486 |
| Guinea | 10,950 | 45,537 | 5,448 | 24,844 | 12,361 | 39,336 | 21,170 | 82,996 | 3,040 | 54,503 |
| Guinea-Bissau | 8,757 | 63,874 | 4,340 | 66,349 | 10,981 | 65,213 | 5,946 | 158,719 | 5,781 | 37,567 |
| Liberia | 6,015 | 54,070 | 6,404 | 78,709 | 8,471 | 32,798 | 18,727 | 405,089 | 16,967 | 76,328 |
| Malawi | 30,389 | 373,393 | 13,620 | 144,304 | 19,794 | 167,011 | 30,989 | 508,482 | 19,033 | 288,792 |
| Mali | 9,597 | 22,859 | 7,055 | 54,394 | 5,341 | 16,115 | 7,623 | 97,376 | 9,817 | 106,541 |
| Mauritania | 13,114 | 49,693 | 4,063 | 18,706 | 10,754 | 51,259 | 20,853 | 97,291 | 10,984 | 89,091 |
| Nepal | 678,640 | 1,637,007 | 483,581 | 1,548,068 | 2,338,457 | 3,717,834 | 4,023,207 | 5,889,145 | 4,037,364 | 4,822,209 |
| Niger | 6,534 | 22,551 | 2,313 | 85,095 | 11,241 | 116,167 | 8,573 | 115,735 | 10,436 | 71,085 |
| Nigeria | 21,092 | 47,766 | 15,634 | 25,588 | 19,455 | 37,621 | 15,409 | 46,386 | 27,558 | 68,635 |
| Republic of Congo | 18,501 | 58,691 | 7,351 | 33,160 | 6,030 | 43,940 | 13,966 | 69,135 | 10,574 | 22,565 |
| Senegal | 5,082 | 22,368 | 5,198 | 140,604 | 9,486 | 109,904 | 31,884 | 259,765 | 20,578 | 321,338 |
| Seychelles | 91,549 | 419,674 | 482,224 | 964,224 | 453,241 | 2,738,501 | 72,998 | 341,221 | 182,684 | 1,424,567 |
| Sierra Leone | 9,071 | 229,161 | 7,754 | 97,707 | 15,325 | 115,967 | 23,337 | 286,750 | 11,751 | 104,044 |
| Sri Lanka | 143,085 | 746,220 | 94,669 | 441,901 | 351,542 | 624,003 | 362,172 | 771,828 | 182,417 | 596,273 |
| Togo | 9,878 | 124,516 | 3,885 | 56,531 | 10,367 | 148,638 | 10,363 | 101,288 | 6,173 | 74,364 |

S1 Table 5: Lines of Credit (LoC): Specific proportions of LoCs that were allocated to health were unavailable. The list of countries receiving LoCs from India is provided below:

| Bangladesh | Mongolia |
| --- | --- |
| Burkina Faso | Mozambique |
| Burundi | Nicaragua |
| Congo | Niger |
| Cote d’ Ivoire | Papua New Guinea |
| Cuba | Rwanda |
| Democratic Republic of Congo | Senegal |
| Ethiopia | Seychelles |
| Fiji | Sierra Leonne |
| Gambia | Sri Lanka |
| Ghana | Sudan |
| Guyana | Suriname |
| Kenya | Tanzania |
| Laos | Uganda |
| Madagascar | Uzbekistan |
| Malawi | Vietnam |
| Maldives | Zambia |
| Mauritania | Zimbabwe |
| Mauritius |  |

S1 Table 6: **Total number of international health sciences students and scholarships**

| Year | Total number of international health sciences students | Estimated number with scholarships |
| --- | --- | --- |
| 2011-12 | 6842 | 1391 |
| 2012-13 | 7767 | 1659 |
| 2013-14 | 8497 | 1744 |
| 2014-15 | 7657 | 1622 |
| 2015-16 | 7784 | 1529 |
| 2016-17 | 7300 | 1613 |
| 2017-18 | 6719 | 1625 |
| 2018-19 | 5839 | 1386 |

We estimated the number of scholarships as part of DAH contributed by India. We used data from the All India Surveys of Higher Education 2011 through 2019 to obtain the number of international students in undergraduate health sciences programs (including medicine, pharmaceutical sciences, dental surgery, and nursing) as well as the total number of international students enrolled by year. We used data on ITECH scholarships to estimate the total number of scholarships offered. Based on these data, we estimated the proportion of total number of students that received scholarships. We assumed these proportions to hold for the total number of health sciences students that receive scholarships. The total number of students enrolled in undergraduate health sciences programs and the estimated number of students with scholarships are reported in below.
