# Supplementary figures and images for: Tracking development assistance for health from India to low- and middle-income countries, 2009–2020
Source: PLoS One. 2022 Dec 12;17(12):e0277799. doi: 10.1371/journal.pone.0277799 (PMC9744314; doi:10.1371/journal.pone.0277799)

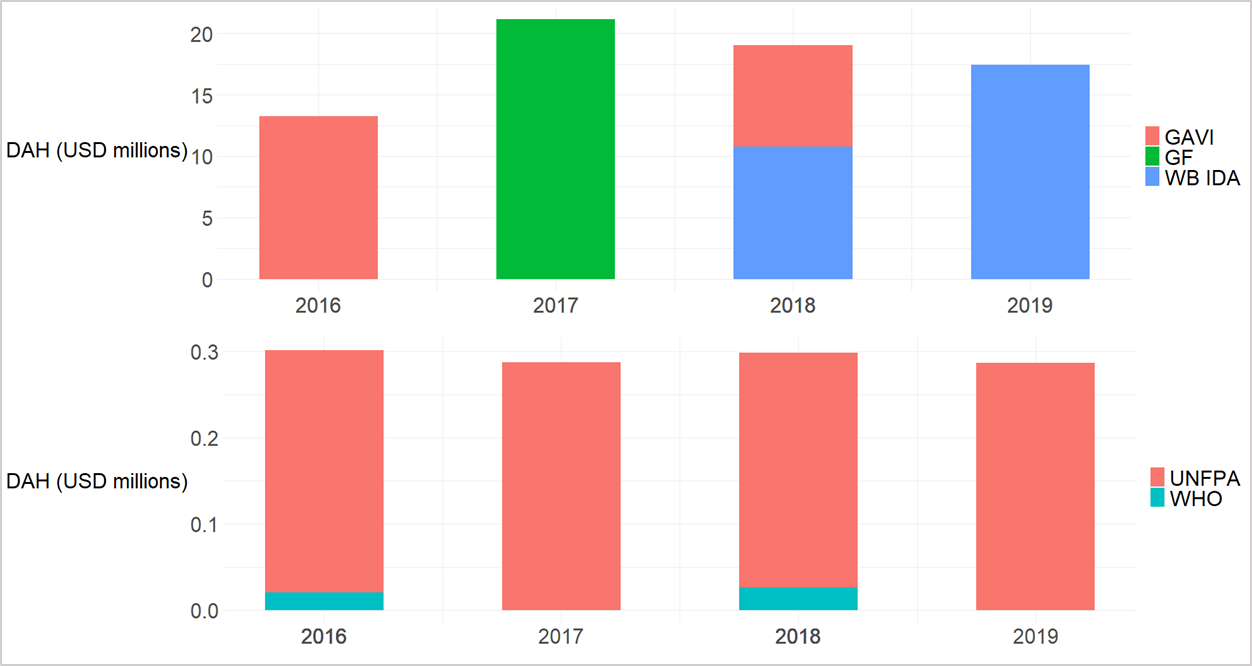

Supplement: S1 Fig — (Note: Multilateral contributions shown here include both annual and replenishment amounts). (TIF) [file pone.0277799.s002.tif]
